# hsa_circ_0111707 Is Associated With Risk of Stress-Related Type 2 Diabetes *via* Sponging miR-144-3p

**DOI:** 10.3389/fendo.2021.790591

**Published:** 2022-01-18

**Authors:** Yu-Xiang Yan, Huan-Bo Xiao, Ya-Ke Lu, Yue Sun, Shuo Wang, Jing Dong, Li-Juan Wu

**Affiliations:** ^1^ Department of Epidemiology and Biostatistics, School of Public Health, Capital Medical University, Beijing, China; ^2^ Municipal Key Laboratory of Clinical Epidemiology, Beijing, China; ^3^ Department of Preventive Medicine, Yanjing Medical College, Capital Medical University, Beijing, China; ^4^ Health Management Center, Xuanwu Hospital, Capital Medical University, Beijing, China

**Keywords:** circRNA, type 2 diabetes, biomarker, neuroendocrine, stress response

## Abstract

**Introduction:**

Chronic stress plays an important role in the development of type 2 diabetes (T2D). Circular RNAs (circRNAs) play significant roles in regulating the pathogenesis of diseases by regulating gene expression. The aim of the present study was to identify the association between hsa_circ_0111707 and stress-related T2D.

**Methods:**

The present study was performed based on a three-part design. The association between hsa_circ_0111707 in peripheral blood mononuclear cells (PBMCs) and T2D and stress-related variables were assessed in a cross-sectional study. The causal relationship of hsa_circ_0111707 on T2D was further investigated in a nested case-control study. miR-144-3p as the miRNA target of hsa_circ_0111707 was verified by dual-luciferase reporter assay and RNA immunoprecipitation (RIP) assay.

**Results:**

The relative expression of hsa_circ_0111707 was significantly lower in the T2D and impaired fasting glucose (IFG) cases in comparison with controls. The hsa_circ_0111707 expression was significantly negatively correlated with miR-144-3p expression and plasma cortisol concentration and positively correlated with *NR3C1* expression. In addition, hsa_circ_0111707 expression was negatively correlated with scores of “demands at work” and “insecurity at work” of Copenhagen Psychosocial Questionnaire (COPSOQ). Decreased hsa_circ_0111707 expression was associated with increased risk of T2D development. Functional analysis demonstrated that hsa_circ_0111707 functions as a sponge for miR-144-3p.

**Conclusion:**

hsa_circ_0111707 is associated with risk of T2D development *via* sponging miR-144-3p. hsa_circ_0111707 in PBMCs can be considered a potential biomarker of stress-related T2D.

## Introduction

Type 2 diabetes (T2D) has become a major public health problem worldwide. In the last decade, the incidence and prevalence of T2D in children and adolescents has increased dramatically (1). In 2015, 415 million people were estimated to have diabetes, more than 90% of whom had type 2 diabetes, with a projected increase to 642 million by 2040 (2). In addition to genetic causes and unhealthful lifestyles, environmental influences, such as psychosocial stress, has been implicated as an important contributor in the development of T2D (3, 4). Psychological stress can affect health through neuroendocrine responses and has been shown to cause impaired glucose homeostasis in earlier experimental and human researches (4, 5). Meta-analytic evidence indicated that job strain, which is defined as high job demands coupled with low control at work, is associated with an increased risk of T2D (6). Association between perceived stress and incident T2D was validated in several cohort studies (7–9). Our previous investigation also showed that chronic stress is associated with insulin resistance (IR), and plasma cortisol is significantly positively correlated with glucose and HOMA-IR (10). Long-term exposure to excess cortisol, such as Cushing syndrome and glucocorticoid treatment, often results in increased susceptibility to hyperglycemia and manifests diabetes (11, 12).

One of the major neuroendocrine systems responding to psychological stress is the hypothalamic-pituitary-adrenal (HPA) axis, with cortisol (glucocorticoid) secretion as its final hormone (13). The HPA axis is highly responsive to the environment, impacts both nervous and other physiological systems, and is critical to health in a wide variety of contexts (14). As a stress hormone, cortisol takes part in the regulation of metabolism, homeostasis, immune reactions, and behavioral responses (15). The effects of cortisol are mediated by intracellular glucocorticoid receptor (GR), coded by the *NR3C1* gene (16). The expression levels of *NR3C1* influence sensitivity to cortisol in physiological and pathophysiological conditions and modify response to cortisol in relevant target tissues (17). Investigation in an occupational population showed that chronic stress is associated with increased plasma cortisol and decreased *NR3C1* expression in circulating monocytes (18). This phenomenon can be explained that reduced *NR3C1* expression helps to alleviate excessive stress-related physiological processes (17).

In the past decade, microRNAs (miRNAs) have been recognized as important regulators of gene expression on the posttranscriptional level through sequence-specific binding to mRNA, which results in inhibiting the translation of protein from mRNA or inducing mRNA degradation (19). miRNA expression levels in rodents and human cells have been found to be altered in response to stress conditions, which in turn alters the expression of HPA axis-related target genes (20). Haramati et al. reported that overexpression of miR-34c appears to reduce cell responsiveness to secrete corticotrophin-releasing hormone (CRH) by inhibiting CRHR1 expression and induce an anxiolytic phenotype (21). miR-135a and miR-124 have also been implicated as potential suppressors of NR3C2 protein expression in rodents (22). In our previous study, let-7b, miR-142, miR-144, and miR-29a have been identified as neuroendocrine stress response-related biomarkers for T2D and IR (23). *NR3C1* was further identified as a target gene of miR-144 (24). These evidences suggest that stress-related miRNAs may be potential novel targets for treatment of stress-related disorders.

Circular RNAs (circRNAs) were recently discovered as a special novel type of endogenous noncoding RNAs (ncRNAs) that are involved in transcriptional and posttranscriptional gene expression regulation (25). circRNAs differ from linear RNAs in that they are circular molecules with covalently closed loop structures and lack 5′ or 3′ polarity or a polyadenylated tail, which makes them much more stable than most linear RNA (26). As the competitive endogenous RNAs (ceRNAs), circRNAs can compete for miRNA-binding sites and make them function as miRNA sponges, thus suppressing miRNA activity and participating in regulation of miRNA target genes (25–27). Accumulated evidence indicates that circRNAs play crucial roles in the onset and progression of diseases and are expected to become diagnostic biomarkers and treatment targets in human diseases (27–29).

Based on differential expression profile of circRNAs in previous microarray analysis (29) and predictive target adsorption relationship between circRNA and miRNA (miRanda), a circulating circRNA, hsa_circ_0111707, was discovered as a potential regulator of *NR3C1* by targeting miR-144-3p. In this study, association between hsa_circ_0111707 expression and T2D was investigated, which may unveil new target for the prevention and treatment of stress-related disorders including T2D.

## Materials and Methods

### Subjects

**Figure 1:**
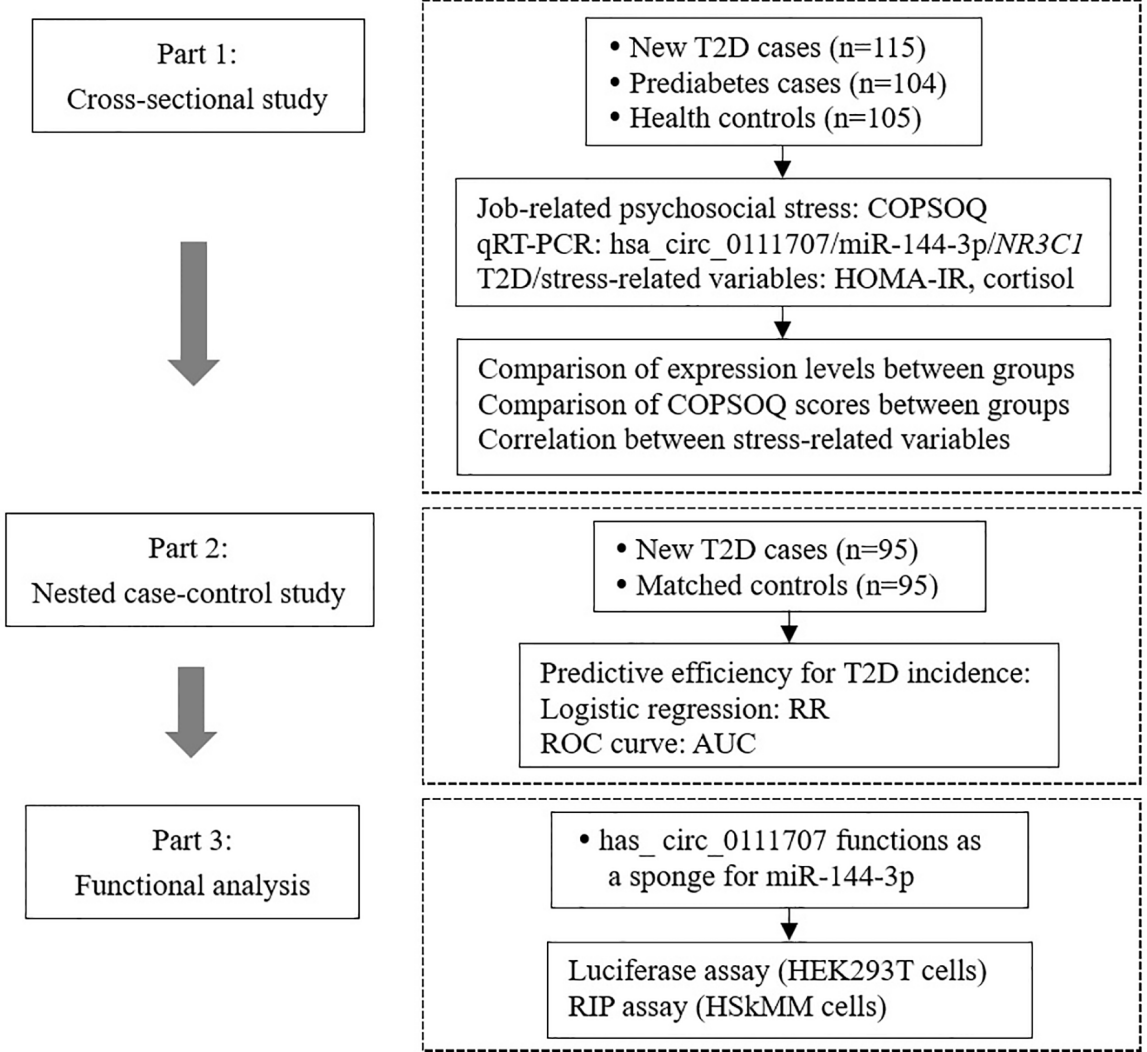
The flowchart of study design. T2D, type 2 diabetes; IFG, impaired fasting glucose; COPSOQ, Copenhagen Psychosocial Questionnaire; HOMA-IR, homeostasis model assessment of insulin resistance; RR, relative risk; AUC, area under the curve; HEK, human embryonic kidney; HSkMM, human skeletal muscle myoblasts.

This study was performed based on a three-part design (**Figure 1**). Firstly, a cross-sectional study was conducted to compare the relative expression of hsa_circ_0111707 and stress-related variables among T2D, prediabetes cases and health controls. In addition, associations between relative expression of hsa_circ_0111707 and stress-related variables were analyzed. Secondly, a nested case-control study, prospectively collecting blood samples from T2D cases and matched controls before clinical diagnosis, was performed to further assess the predictive efficiency of hsa_circ_0111707 for T2D development. Thirdly, luciferase assay and RNA immunoprecipitation (RIP) assay were used to validate the function of hsa_circ_0111707 as a sponge for miR-144-3p.

In the first part of the study, the participants were composed of 324 individuals, including 115 newly diagnosed T2D cases, 105 health controls, and 104 individuals with impaired fasting glucose (IFG) as prediabetes cases. Controls and IFG cases were frequency matched with T2D cases according to gender and age (± 3 years). All the participants were aged from 30 to 65 and were recruited from January 2016 to January 2017 in a functional community cohort composed of nearly 10,000 employees in Beijing Xicheng district (23). In the second part of the study, cases were newly diagnosed T2D patients in the same cohort during 2020. A total of 95 incident T2D cases were identified. Controls were selected from study participants who did not develop T2D during the follow-up period. For each case, one control was matched with the criteria of gender and age (± 12 months). The relative expression of hsa_circ_0111707 at baseline in 2016 between the two groups was compared. The average time between blood collection and date of diagnosis was 4 years.

The diagnosis of T2D and IFG were in accordance with the 1999 World Health Organization (WHO) criteria (30). T2D: fasting plasma glucose (FPG) levels ≥7.0 mmol/L or 2 h glucose levels ≥11.1 mmol/L in oral glucose tolerance test (OGTT). IFG: 6.1 ≤ FPG <7.0 mmol/L. Patients with a past history of using antidiabetic treatment or any endocrine disease other than T2D, were excluded from the study. Other exclusion criteria were described in previous study (23). This study was approved by the university ethical committee, and informed consent was obtained from each participant.

### Data Collection

All subjects attended a standardized examination protocol. A structured questionnaire was used to collect information including demographic data, medical history, current medication, and health-related behaviors (10). The Chinese translation and adaptation of the short standard version of the Copenhagen Psychosocial Questionnaire (COPSOQ) was used to assess job-related psychosocial stress (10, 31, 32). The COPSOQ version used in this study comprises 5 scales including 34 items concerning (1) demands at work, (2) work organization and content, (3) interpersonal relations and leadership, (4) insecurity at work, and (5) job satisfaction. All scales had a 5-point Likert format (except scales measuring insecurity at work), reflecting either intensity or frequency. These scales were transformed into a theoretical range, extending from 0 (do not agree at all) to 100 (fully agree) points. The higher score for scales of “demands at work” and “insecurity at work” and lower score for scales of “work organization and content”, “interpersonal relations and leadership”, and “job satisfaction” indicate higher level of job strain.

### Blood Sample Collection and RNA Extraction

Following an at least 8 h overnight fast, venous blood samples were collected in EDTA tubes from each subject between 7:30 and 8:30 a.m. in a calm state; 2 ml of the blood samples were immediately centrifuged to retrieve plasma. Another 2 ml blood samples were used to isolate peripheral blood mononuclear cells (PBMCs) by density gradient centrifugation. Total RNA was extracted from PBMCs using TRIzol reagent (Invitrogen, Waltham, MA, USA), following the manufacturer’s instructions. The purity and concentration of extracted RNA were determined by Nano Drop 2000 spectrophotometer, and the integrity was inspected by Agilent Bioanalyzer 2100 (Agilent Technologies, Santa Clara, CA, USA).

### Anthropometric Measurement and Biochemical Analysis

Anthropometric parameters including weight, height, waist circumference (WC), and blood pressure were obtained using standard measurement (26). Body mass index (BMI) was calculated as weight (kg)/height (m)^2^. FPG, glycated hemoglobin (HbAlc), triglyceride (TG), total cholesterol (TCH), high-density lipoprotein cholesterol (HDLC), and low-density lipoprotein cholesterol (LDLC) were tested through standard procedures in the clinical laboratory of the hospital (23). Plasma concentrations of cortisol and insulin were measured by radioimmunoassay (RIA) kit. The homeostasis model assessment index of insulin resistance (HOMA-IR) calculated as “fasting insulin (μIU/ml) × fasting glucose (mmol/L)/22.5.”

### Quantitative Real-Time PCR

The expression levels of hsa_circ_0111707, miR-144-3p, and *NR3C1* were measured using quantitative real-time PCR (qRT-PCR). RNA was reverse transcribed into cDNA with PrimeScript RT Reagent Kit (Takara, Beijing, China) and miScript Reverse Transcriptase Kit (Qiagen, Hilden, Germany), respectively, according to manufacturer’s instructions. The real-time PCR analyses were performed using SYBR Premix Ex TaqII (Takara) with LightCycler 480 II Real-Time PCR Instrument (Roche, Basel, Switzerland). β-Actin was used as an internal reference for quantification of circRNA and mRNA, while miR-451a for miRNA (miR-451a expression was stable and consistent throughout all the evaluated samples) (23). The specific primers used are listed in **Supplementary Table S1**. The relative expression of genes was calculated by 2^−ΔΔCT^ method.

### Cell Culture

Human embryonic kidney 293T (HEK293T) cells were cultured in Dulbecco’s modified Eagle’s medium (DEME, HyClone, Logan, UT, USA) containing 10% fetal bovine serum (FBS). Human skeletal muscle myoblasts (HSkMM, ScienCell, Carlsbad, CA, USA) were cultured with Skeletal Muscle Cell Medium (Cat. #3501, ScienCell, USA). All these cell lines were maintained at 37°C with 5% CO_2_ in a humidified incubator.

### Luciferase Reporter Assay

The full length of 346 bp of hsa_circ_0111707 and its corresponding mutant version without miR-144-3p binding site were constructed and inserted downstream of the luciferase reporter gene of the plasmid psiCHECK2 (The fragment sequences were listed in **Supplementary Table S2**). All these plasmids were validated by sequencing. HEK293T cells (1 × 10^5^ cells/well) were seeded in 96-well plates overnight and transfected with a luciferase reporter plasmid and miR-144-3p mimic or negative control (NC) using the Lipofectamine 2000 transfection reagent (Invitrogen), whereas empty psiCHECK2 was used as control plasmid. After 48 h, the activities of firefly and Renilla luciferase were detected with dual-luciferase reporter assay Kit (Promega, Madison, WI, USA). The relative activity was equal to firefly luciferase activity/Renilla luciferase activity. All experiments were independently in triplicate.

### RNA Immunoprecipitation Assay

Skeletal muscle is an important peripheral tissue that utilizes blood glucose. Cortisol impairs glucose uptake in peripheral tissues like muscle and fat, which may facilitate insulin resistance (33). To further support the connection between circRNA and miRNA in skeletal muscle cells, the RIP assay was performed by a Magna RIP Kit (Millipore, Burlington, MA, USA) according to the manufacturer’s methods. HSkMM cells were transfected with miR-144-3p mimics or miR-NC, and then lysed in complete RNA lysis buffer after 48 h. Cell lysates were divided into three fractions. Two of them then incubated with magnetic beads which were conjugated with anti-Argonaute2 (AGO2) or negative control IgG antibody overnight at 4°C. Immunoprecipitated RNAs were extracted to detect the target RNAs including miR-144-3p and hsa_circ_0111707 expressions by RT-qPCR. The other one fraction was used as blank control (input).

### Statistical Analysis

The data were represented as the mean ± standard deviation (SD) or percentages when they fit. The normal distribution of the data was checked using the Kolmogorov-Smirnov test. Student’s *t*-test or one-way analysis of variance (ANOVA) were performed to examine the differences between the study groups if the data obeyed normal distribution, otherwise nonparametric test (Mann-Whitney *U* test or Kruskal-Wallis *H* test) was used. Chi-square test was used to compare the categorical data among groups. Spearman’s correlation coefficient was used to evaluate the relationship between relative expression of hsa_circ_0111707 and stress-related variables. The association between circRNA expression and risk of T2D was analyzed using covariate-adjusted logistic regression and relative risk (RR). Receiver operator characteristic (ROC) curve was used to identify the best cut-off value of circRNA expression to predict T2D. Statistical analyses were performed by using SPSS 24.0, GraphPad Prism 5.01 and R 3.5.1. The level of statistical significance was set at two-sided *P*< 0.05.

## Results

### Basic Characteristics of the Participants in the First Part of the Study

The demographic and clinical characteristics of the study participants in the first part of the study (the cross-sectional study) are presented in **Table 1**. There was no significant difference in the distribution of age, gender, smoking, alcohol use, and physical inactivity among the three groups. The average levels of most of the clinical parameters, including BMI, WC, TCH, TG, LDLC, HDLC, and HOMA-IR, were significantly different among the three compared groups (*p* < 0.05).

**Table 1:** Demographic and clinical characteristics of study participants in the first part of the study.

Variable	T2D (*n* = 115)	IFG (*n* = 104)	Control (*n* = 105)	*p*-value
Age (years)	54.91 ± 7.46	54.08 ± 7.77	53.97 ± 8.13	0.651^c^
Gender (male/female)	60/55	55/49	55/50	0.994^b^
BMI (kg/m^2^)	27.00 ± 3.05^d^	26.64 ± 3.15^d^	23.89 ± 3.12	<0.001^a^
WC (cm)	89.29 ± 8.56^f^	86.95 ± 9.44^f^	82.06 ± 9.71	<0.001^c^
SBP (mmHg)	133.01 ± 16.55^d,e^	128.07 ± 18.61	124.04 ± 17.09	0.001^a^
DBP (mmHg)	82.24 ± 10.74^d,e^	79.00 ± 9.95	76.33 ± 9.66	<0.001^a^
TCH (mmol/L)	5.29 ± 0.93^d,e^	4.98 ± 1.06	4.84 ± 0.94	0.003^a^
TG (mmol/L)	2.39 ± 1.64^f,g^	1.93 ± 1.21^f^	1.62 ± 1.38	<0.001^c^
LDLC (mmol/L)	3.21 ± 0.91^f,g^	2.92 ± 0.74	2.83 ± 0.56	0.001^c^
HDLC (mmol/L)	1.48 ± 0.37^f^	1.51 ± 0.26	1.57 ± 0.28	0.001^c^
FPG (mmol/L)	9.26 ± 2.30^f,g^	6.49 ± 0.25^f^	4.97 ± 0.40	<0.001^c^
HbA1c (%)	7.51 ± 1.40^f,g^	5.74 ± 0.60^f^	5.20 ± 0.42	<0.001^c^
Insulin (uIU/ml)	15.01 ± 2.83^f,g^	12.79 ± 2.51^f^	10.07 ± 2.95	<0.001^c^
HOMA-IR	6.18 ± 1.79^d,e^	3.67 ± 0.77^d^	2.23 ± 0.71	<0.001^a^
Cortisol (ng/ml)	213.85 ± 39.14^f^	202.72 ± 35.74^f^	174.44 ± 31.27	<0.001^c^
Smoking (*n*, %)	18, 15.65	16, 15.38	11, 10.48	0.469^b^
Alcohol use (*n*, %)	12, 10.43	13, 12.50	8, 7.62	0.503^b^
Physical activity (*n*, %)	83, 72.17	74, 71.15	85, 80.95	0.197^b^

T2D, type 2 diabetes; IFG, impaired fasting glucose; BMI, body mass index; WC, waist circumference; SBP, systolic blood pressure; DBP, diastolic blood pressure; TCH, total cholesterol; TG, triglyceride; LDLC, low-density lipoprotein cholesterol; HDLC, high-density lipoprotein cholesterol; FPG, fast plasma glucose; HbA1c, glycated haemoglobin; HOMA-IR, homeostasis model assessment of insulin.

a One-way ANOVA.

b Chi-square test.

c Kruskal-Wallis H test.

d Significantly different from control group (p < 0.05 with LSD test).

e Significantly different from IFG group (p< 0.05 with LSD test).

f Significantly different from control group (p < 0.05 with Mann-Whitney U test).

g Significantly different from IFG group (p < 0.05 with Mann-Whitney U test).

As to the COPSOQ investigation, those with abnormal FPG, including T2D and IFG, showed a higher level of psychosocial stress than those with normal FPG (**Table 2**). Compared with the control group, the average scores on scales of demands at work and insecurity at work were significantly higher in the T2D and IFG groups (*p* < 0.05). However, no significant differences of the average scores of these two scales were observed between T2D and IFG groups.

**Table 2:** Association between COPSOQ variables and T2D.

Variables	T2D group	IFG group	Control	*p*-value^*^
**Demands at work**	57.54 ± 19.20^a^	55.01 ± 13.87^a^	49.01 ± 18.91	0.001
Work organization and content	62.43 ± 17.79	62.02 ± 16.55	60.24 ± 16.05	0.597
Interpersonal relations and leadership	58.33 ± 17.65	62.28 ± 20.23	60.57 ± 18.37	0.293
**Insecurity at work**	56.74 ± 41.67^a^	52.40 ± 40.10^a^	39.29 ± 41.15	0.005
Job satisfaction	59.08 ± 20.72	59.68 ± 23.16	61.31 ± 21.25	0.736

COPSOQ, Copenhagen Psychosocial Questionnaire; T2D, type 2 diabetes; IFG, impaired fasting glucose.

^*^One-way ANOVA.

a Significantly different from control group (p < 0.05).

### Comparison of Relative Expression of hsa_circ_0111707, miR-144-3p, and NR3C1 Among the Study Groups

**Figure 2:**
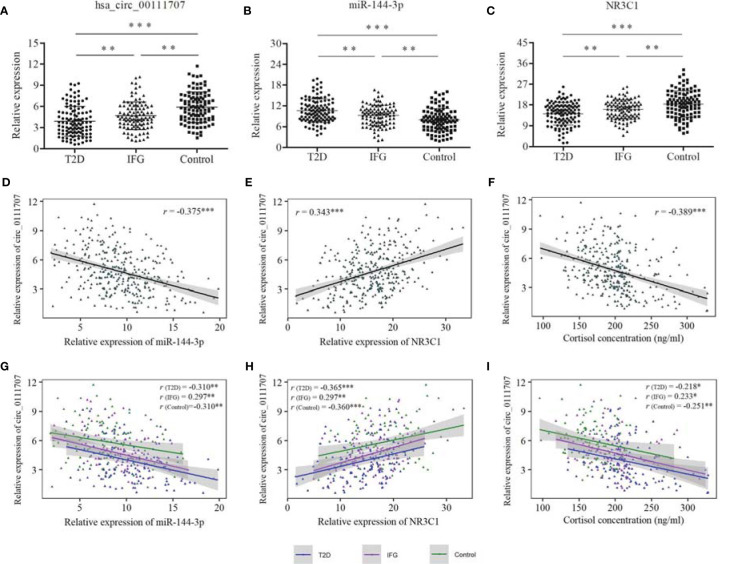
Relative expression of hsa_circ_0111707, miR-144-3p, and *NR3C1* and Spearman’s correlation between hsa_circ_0111707 and miR-144-3p, *NR3C1*, and cortisol concentration. **(A–C)** Comparison between T2D, IFG, and control groups. **(D–F)** Spearman’s correlation between relative expression of hsa_circ_0111707 and miR-144-3p, *NR3C1*, and cortisol concentration in all the subjects. **(G–I)** Spearman’s correlation between relative expression of hsa_circ_0111707 and miR-144-3p, *NR3C1*, and cortisol concentration in subgroups. T2D, type 2 diabetes; IFG, impaired fasting glucose. ^*^ *p* < 0.05, ^**^ *p* < 0.01, ^***^ *p* < 0.001.

The relative expression of hsa_circ_0111707, miR-144-3p, and *NR3C1* in PBMCs and plasma concentration of cortisol showed significantly differences among the T2D, IFG and control groups (**Figures 2A–C**; **Table 1**). Fold changes (FCs) of expression of the compared groups were also analyzed and presented in **Supplementary Table S3**. The relative expression of hsa_circ_0111707 and *NR3C1* were significantly lower in the T2D and IFG cases in comparison with the controls, being lower in the T2D cases in comparison with IFGs (**Figures 2A, C** **)**. By contrast, as the miRNA target of hsa_circ_0111707 and stress hormone, respectively, relative expression of miR144-3p and plasma cortisol concentration were significantly higher in the T2D and IFG cases in comparison with the controls, being higher in the T2D cases in comparison with IFGs (**Figure 2B** and **Table 1**).

### The Relationship Between hsa_circ_0111707 Expression and Stress-Related Variables

The Spearman’s correlation analysis revealed that the relative expression of hsa_circ_0111707 was significantly negatively correlated with miR-144-3p and plasma cortisol concentration and positively correlated with the relative expression of *NR3C1* (**Figures 2D–F**). In subgroup analysis, significant correlations were also observed between hsa_circ_0111707 and miR-144-3p, *NR3C1*, and cortisol in the T2D, IFG, and control groups, respectively (**Figures 2G–I**). At the same time, significant correlations also existed between miR-144-3p, *NR3C1*, and cortisol, in all the subjects and subgroups (**Supplementary Table S4**), which indicated the potential regulation relationship among them. Relative expression of *NR3C1* was negatively associated with miR-144-3p and cortisol concentration, while positive association existed between miR-144-3p and cortisol (**Supplementary Table S4**).

The relationship between relative expression of hsa_circ_0111707 and job-related psychosocial stress assessed by COPSOQ was also analyzed, which showed that relative expression of hsa_circ_0111707 was negatively correlated with scores on scales of demands at work and insecurity at work. Whereas, plasma cortisol concentration was positively correlated with scores of demands at work and insecurity at work (**Table 3**).

**Table 3:** Spearman’s correlation matrix of hsa_circ_ 0111707 expression and COPSOQ variables.

Scales	1.	2.	3.	4.	5.	6.	7
1. **hsa_circ_0111707**	1.00						
2. Cortisol	0.39^*^	1.00					
3. **Demands at work**	−0.23^*^	0.21^*^	1.00				
4. Work organization and content	−0.04	0.04	−0.10	1.00			
5. Interpersonal relations and leadership	0.10	−0.04	−0.14^*^	0.47^*^	1.00		
6. **Insecurity at work**	−0.13^*^	0.16^*^	0.43^*^	0.03	−0.03	1.00	
7. Job satisfaction	0.13^*^	−0.08	−0.24^*^	0.45^*^	0.55^*^	−0.13^*^	1.00

COPSOQ, Copenhagen Psychosocial Questionnaire.

^*^p < 0.05.

### The Relationship Between hsa_circ_0111707 Expression and HOMA-IR

As insulin resistance is the important pathophysiological basis of T2D, the relationship between relative expression of hsa_circ_0111707 and HOMA-IR was assessed. Significantly, negative correlations were observed between hsa_circ_0111707 expression and HOMA-IR in all the subjects (*r* = −0.447, *p* < 0.001), T2D (*r* = −0.278, *p* = 0.003), and IFG groups (*r* = −0.203, *p* = 0.039). Multiple linear regression analysis was further performed to confirm the association between hsa_circ_0111707 expression and HOMA-IR, with potential confounders being adjusted (**Supplementary Table S5**). The results showed that the relative expression of hsa_circ_0111707 was a significant negative predictor of HOMA-IR (*p* < 0.001), indicating that decreased hsa_circ_0111707 expression may contribute to insulin resistance. If BMI was replaced by WC among the above covariables in the model, hsa_circ_0111707 expression was still associated with HOMA-IR independent of obesity index.

### The Association Between hsa_circ_0111707 Expression and Risk of T2D

**Figure 3:**
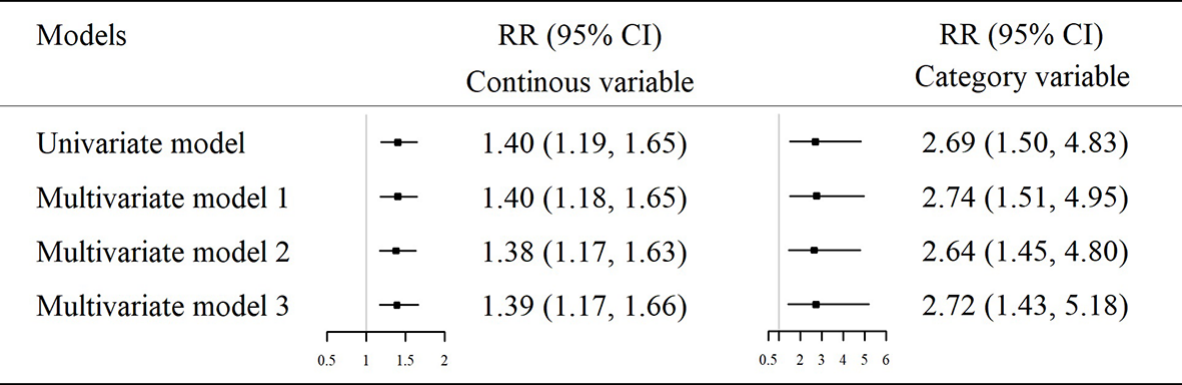
The association between relative expression of hsa_circ_0111707 assessed by univariate and multiple logistic regression analyses. Model 1: adjusted for smoking, drinking, and physical activity. Model 2: further adjusted for BMI based on model 1. Model 3: further adjusted for TC, TG, HDLC, LDLC, SBP, and DBP based on model 2. RR, relative risk; CI, confidence interval; T2D, type 2 diabetes; IFG, impaired fasting glucose.

In the second part of the study (nested case-control study), the demographic and clinical characteristics of the newly diagnosed T2D cases and their matched controls at 4 years prior to the diagnosis (baseline) were presented in **Supplementary Table S6**. The relative expression of hsa_circ_0111707 in the T2D cases (4.99 ± 1.69) was significantly lower than the controls (6.21 ± 2.11, *p* <0.001). The adjusted RR for the per-unit decrease of hsa_circ_0111707 expression was 1.38 (95% CI: 1.17–1.63) in the fully adjusted model (**Figure 3**). When hsa_circ_0111707 expression was grouped according to the median (5.375), participants with lower expression of hsa_circ_0111707 had a 2.72-fold (95% CI: 1.43–5.18) increased risk for T2D, compared with those with higher expression level (**Figure 3**). The ROC analyses showed that the area under curve (AUC) was 0.667 (95% CI: 0.590–0.744) for hsa_circ_0111707 (*p* < 0.001). The relative expression value of 5.845 was identified as the best cutoff point of hsa_circ_0111707 to predict T2D (sensitivity: 0.716, specificity: 0.568).

### hsa_circ_0111707 Functions as a Sponge for miR-144-3p

**Figure 4:**
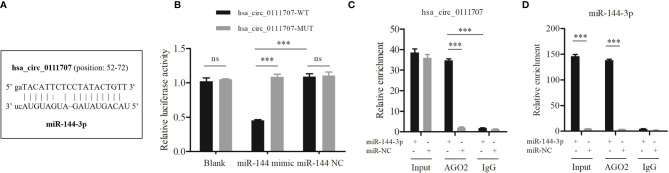
hsa_circ_0111707 functions as a sponge for miR-144-3p. **(A)** The binding sites of hsa_circ_0111707 and miR-144-3p. **(B)** Luciferase assays revealed that miR-144-3p is a miRNA target of hsa_circ_0111707 in HEK293T. **(C, D)** Anti-AGO2 RIP was executed in HSkMM cells after transfection with miR-144-3p mimic or miR-NC, followed by qRT-PCR to detect the enrichment (expression) of hsa_circ_0111707 and miR-144-3p, respectively. HEK, human embryonic kidney; HSkMM, human skeletal muscle myoblasts; WT, wild type; MUT, mutant; Blank: empty plasma; Input: blank control. ^***^ *p* < 0.001. ns, not significant.

The luciferase assays showed that the average relative luciferase activity of cells cotransfected with miR-144-3p and hsa_circ_0111707-WT was significantly lower than that in cells cotransfected with miR-144-3p and hsa_circ_0111707-MUT (*p* < 0.001, **Figures 4A, B**). It has been known that miRNAs regulate target gene expression by binding to AGO2, the key component of RNA-induced silencing complex (RISC). Thus, an anti-AGO2 RIP assay was conducted in HSkMM cells to pull down the RNA transcripts which bind to AGO2 with anti-AGO2 antibody and IgG as a NC. The results showed that both hsa_circ_0111707 and miR-144-3p were significantly immunoprecipitated by anti-AGO2 antibody compared with IgG and enriched by miR-144-3p mimics compared with miR-NC (**Figure 4C, D** **)**. These data demonstrated that hsa_circ_0111707 acts as a ceRNA to sponge miR-144-3p by directly binding to it.

## Discussion

The present study identified the association between relative expression of hsa_circ_0111707 in PBMCs and T2D in an occupational cohort. Functional analysis demonstrated that hsa_circ_0111707 acts as a sponge for miR-144-3p, which is a direct regulator of *NR3C1*.

In this study, a valid and relevant short version of the COPSOQ instrument, which covers a broad range of aspects of currently leading concepts and theories (① the job characteristics model, ② the Michigan organizational stress model, ③ the demand-control-support model, ④ the sociotechnical approach, ⑤the action-theoretical approach, ⑥the effort-reward-imbalance model, ⑦the vitamin model), was used to assess psychosocial stress at work (31 24). The COPSOQ is a comprehensive instrument for the assessment of psychosocial factors at work. The main advantage of the COPSOQ is its generic usability in all sorts of professions and industries. The user-friendly version of the short COPSOQ, which almost reaches the same level of criterion validity as the long version, is more suitable for large-scale survey (34).

We found that subjects with abnormal increased FPG showed a higher level of work-related psychosocial stress than those with normal FPG. The mean scores of demands at work and insecurity at work” of the COPSOQ were significantly higher in the T2D and IFG groups than those in the control group, suggesting that psychosocial stress is associated with T2D. The significant association observed between COPSOQ and cortisol was consistent with the concept that, as a stimulus, psychological stress activates the HPA axis and results in cortisol rise (13). Decreased relative expression of *NR3C1* in PBMCs and increased plasma cortisol concentration in T2D and IFG cases further support the association between psychological stress and T2D. The negative correlation between relative expression of *NR3C1* and cortisol concentration can be explained that reduced *NR3C1* expression helps to alleviate excessive stress-related physiological processes (17).

In our previous study, *NR3C1* was proved to be a direct target gene of miR-144-3p by luciferase assay (24). miR-144 has been identified as a stressor-responsive miRNA by several studies. A human miRNA microarray and subsequent RT-qPCR confirmed that elevated miR-144 in whole blood is a potential biomarker for naturalistic stress in healthy young adults (35). miR-144 has also been reported as a potential signature miRNA for detection of T2D based on the evidence that miR-144 expression was highly upregulated in T2D, and it seemed to exhibit a linear relationship with increasing glycemic status and insulin resistance (23, 36). We found that significantly increased miR144-3p expression and decreased *NR3C1* expression in PBMCs among subjects with abnormal increased FPG, and significantly negative correlation between them, which support the relationship that miR-144-3p negatively regulator of *NR3C1*.

We found that the relative expression of hsa_circ_0111707 significantly decreased in the PBMCs in T2D and IFG cases. At the same time, significant negative relationship existed between hsa_circ_0111707 expression and scores of demands at work and insecurity at work. miR-144-3p was further identified as a miRNA target of hsa_circ_0111707 in this study. We inferred that decreased hsa_circ_0111707 expression might increase the level of target miRNA and miRNA hyper expression promotes degradation of downstream target gene (by binding to 3-untranslated region of mRNA), thereby affecting the susceptibility of T2D development. At population level, the relative expression of hsa_circ_0111707 was significantly negatively correlated with miR-144-3p and cortisol concentration, and positively correlated with expression of *NR3C1*. These results were in conjunction with our hypothesis that hsa_circ_0111707 can compete for miRNA-binding sites and make it function as miR-144-3p sponge, thus suppressing miR-144-3p activity and participating in regulation of downstream target gene *NR3C1* of miR-144-3p.

Based on the cross-sectional study, the predictive efficiency of hsa_circ_0111707 for T2D was further investigated in a nested case-control study, which allows us to estimate the causal relationship of hsa_circ_0111707 on the development of T2D. The measurement of hsa_circ_0111707 expression and other potential risk factors were prior to the diagnosis of T2D. The decreased hsa_circ_0111707 expression 4 years prior to the diagnosis of T2D was associated with increased risk of T2D development. hsa_circ_0111707 expression was an independent predictor of T2D, and insulin resistance with covariates was adjusted. The best cutoff value of hsa_circ_0111707 expression to predict T2D was also identified using ROC analysis.

Over the past few years, accruing evidence has revealed ncRNAs including microRNAs, long noncoding RNAs, and circular RNAs regulate pivotal cellular and molecular processes during all stages of a number of diseases (37). It is known that circulating ncRNAs are involved in cell-to-cell communication and correlated with the expression of target mRNAs in pathways that important to the development of disease (29, 38). The alternation expression of these ncRNAs in circulation has been demonstrated as valuable biomarkers in clinical practices (39, 40). As a window, PBMCs convert psychosocial stress into cellular dysfunction and finally contribute to the pathophysiology of lifestyle-related diseases such as T2D, cardiovascular disease, and atherosclerosis (41). In the present study, by identification of the association between hsa_circ_0111707 and stress-related T2D, it would be helpful for discovering novel targets for the prevention, prediction, and more personalized treatment of neuroendocrine stress-related disorders. Our results are reliable based on the three-part design. However, there are several limitations in our study. Firstly, the present study was performed in an occupational cohort, which affects the extrapolation of the results. Further prospective studies with larger sample sizes are needed to confirm our findings. Secondly, experimental studies are needed to further unveil the molecular mechanisms of the roles of hsa_circ_0111707 in T2D development.

In conclusion, our findings suggest that hsa_circ_0111707 is associated with risk of T2D development and may be involved in downregulation of *NR3C1 via* sponging miR-144-3p. hsa_circ_0111707 in PBMCs can be considered a potential biomarker of stress-related T2D.

## Data Availability Statement

The raw data supporting the conclusions of this article will be made available by the authors, without undue reservation.

## Ethics Statement

The studies involving human participants were reviewed and approved by the Capital Medical University ethical committee. The patients/participants provided their written informed consent to participate in this study.

## Author Contributions

Y-XY designed the study and drafted the manuscript. H-BX, YS, JD, and L-JW collected the data and samples. Y-XY and L-JW conducted statistical analyses. Y-XY, YS, and SW conducted the experiments. All authors interpreted the data and have approved the final manuscript.

## Funding

This study was supported by the National Natural Science Foundation (81773511, 81573214) and the Beijing Municipal Natural Science Foundation (7162020).

## Conflict of Interest

The authors declare that the research was conducted in the absence of any commercial or financial relationships that could be construed as a potential conflict of interest.

## Publisher’s Note

All claims expressed in this article are solely those of the authors and do not necessarily represent those of their affiliated organizations, or those of the publisher, the editors and the reviewers. Any product that may be evaluated in this article, or claim that may be made by its manufacturer, is not guaranteed or endorsed by the publisher.

## Abbreviations

AUC, area under the curve; BMI, body mass index; circRNA, circular RNA; ceRNA: competitive endogenous RNA; COPSOQ, Copenhagen Psychosocial Questionnaire; DBP, diastolic blood pressure; FBS, fetal bovine serum; FPG, fasting plasma glucose; FC, fold change; HbA1c, glycated hemoglobin; HDLC, high-density lipoprotein-cholesterol; HEK, human embryonic kidney; HSkMM, human skeletal muscle myoblasts; HOMA-IR, homeostasis model assessment of insulin resistance; IFG, impaired fasting glucose; IR, insulin resistance; LDLC, low-density lipoprotein-cholesterol; miRNA, microRNA; MUT, mutant; NC, negative control; ncRNA, noncoding RNA; PBMC, peripheral blood mononuclear cell; ROC, receiver operating characteristic curve; RR, relative risk; qRT-PCR, real-time quantitative polymerase chain reaction; RIP: RNA immunoprecipitation; SBP, systolic blood pressure; TCH, total cholesterol; TG, triglyceride; T2D, type 2 diabetes; WC, waist circumference; WT, wide type.
