# Supplementary material for: hsa_circ_0111707 Is Associated With Risk of Stress-Related Type 2 Diabetes via Sponging miR-144-3p
Source: Front Endocrinol (Lausanne). 2022 Jan 18;12:790591. doi: 10.3389/fendo.2021.790591 (PMC8803902; doi:10.3389/fendo.2021.790591)
Supplement: Supplementary file 1 [file DataSheet_1.docx]

**Supplementary Data**

Table S1. The primer sequences

| RNA | Primer sequences |
| --- | --- |
| β-actin | F: 5' ACTTAGTTGCGTTACACCCTT 3'  R: 5' CCACAGCCAGCAACAAGTC 3' |
| hsa_circ_0111707 | F: 5' GGCTCAGTGAAGGCCATCAT 3'  R: 5' CCACAGCCAGCAACAAGTC 3' |
| *NR3C1* | F: 5' AAAACTATGGGAGGAAACC 3'  R: 5' GGAGTGACCAGCCAAGATG 3' |
| miR-144-3p | 5′ TACAGTATAGATGATGTACT 3′ |
| miR‐451a | 5′ AAACCGTTACCATTACTGAGTT 3′ |

Table S2. The sequences of circRNA and miRNA fragments in the constructed plasmids

| Fragments | Sequences |
| --- | --- |
| hsa_circ_0111707-346bp-wt | ATTAAAGTATGGACAGCCAACCCCCAACAATTTGTAGAAGATGAAGATGATGATACATTCTCCTATACTGTTAGAATAGCAGCTCAAGACTTGTTGCTGGCTGTGGCCACAGATTTCCAGAATGAAAGTGCAGCAGCCCTGGCTGCTGCAGCCACTCGACATTTACAAGAAGCTGAGCAAACCAAAAACAGTGGCACTGAGCACTGGTGGAAGATCCATGAGGCATGCATGCTTGCCCTAGGCTCAGTGAAGGCCATCATCACTGACAGTGTGAAAAATGGCAGGATTCATTTTGACATGCATGGGTTCCTGACCAATGTCATCCTTGCAGACCTCAACCTCTCAG |
| hsa_circ_0111707-346bp-mut | ATTAAAGTATGGACAGCCAACCCCCAACAATTTGTAGAAGATGAAGATGATGAATGTATCACGATATGACATAGAATAGCAGCTCAAGACTTGTTGCTGGCTGTGGCCACAGATTTCCAGAATGAAAGTGCAGCAGCCCTGGCTGCTGCAGCCACTCGACATTTACAAGAAGCTGAGCAAACCAAAAACAGTGGCACTGAGCACTGGTGGAAGATCCATGAGGCATGCATGCTTGCCCTAGGCTCAGTGAAGGCCATCATCACTGACAGTGTGAAAAATGGCAGGATTCATTTTGACATGCATGGGTTCCTGACCAATGTCATCCTTGCAGACCTCAACCTCTCAG |
| has-miR-144-3p | sense (5'-3'): UACAGUAUAGAUGAUGUACU  antisense (5'-3'): UACAUCAUCUAUACUGUAUU |
| miRNA NC | sense (5'-3'): UUCUCCGAACGUGUCACGUTT  antisense (5'-3'): ACGUGACACGUUCGGAGAATT |

Table S3. Fold changes of gene expression of the compared groups

| Variables | T2D *vs*. Control | |  | IFG *vs*. Control | |  | T2D *vs*. IFG | |
| --- | --- | --- | --- | --- | --- | --- | --- | --- |
|  | FC^a^ | *P* |  | FC^a^ | *P* |  | FC^a^ | *P* |
| hsa_circ_0111707^b^ | 0.615 | <0.001 |  | 0.759 | <0.001 |  | 0.811 | 0.002 |
| miR-144-3p^c^ | 1.318 | <0.001 |  | 1.240 | 0.004 |  | 1.063 | 0.002 |
| *NR3C1*^c^ | 0.804 | <0.001 |  | 0.893 | 0.001 |  | 0.900 | 0.005 |

T2D, type 2 diabetes; IFG, impaired fasting glucose; FC, Fold change.

^a^ Fold change was analyzed by calculating the ratio of the median of the variables in the two compared groups. ^b^ Mann-Whitney U test. ^c^ least significant difference (LSD) test following one-way ANOVA.

Table S4. Spearman correlation among the relative expression of miR-144-3p, *NR3C1* and cortisol concentration

|  | *NR3C1* | Cortisol |
| --- | --- | --- |
| All the subjects |  |  |
| miR-144-3p | -0.342^**^ | 0.275^**^ |
| NR3C1 | 1.000 | -0.334^**^ |
| T2D group |  |  |
| miR-144-3p | -0.283^**^ | 0.251^**^ |
| NR3C1 | 1.000 | -0.311^**^ |
| IFG group |  |  |
| miR-144-3p | -0.285^**^ | 0.177 |
| NR3C1 | 1.000 | -0.293^**^ |
| Control group |  |  |
| miR-144-3p | -0.247^*^ | 0.051 |
| NR3C1 | 1.000 | -0.017 |

^*^*P*<0.05, ^**^*P*<0.01.

Table S5. Stepwise multiple linear regression analysis of the relationship between hsa_circ_0111707 expression and HOMA-IR

| Variables | Adjusted for BMI | |  | Adjusted for WC | |
| --- | --- | --- | --- | --- | --- |
|  | *β* coefficient | *P* |  | *β* coefficient | *P* |
| BMI/WC | 0.093 | 0.022 |  | 0.042 | 0.003 |
| TG | 0.380 | <0.001 |  | 0.385 | <0.001 |
| LDLC | 0.081 | 0.032 |  | 0.084 | 0.041 |
| hsa_circ_0111707 | -0.467 | <0.001 |  | -0.470 | <0.001 |

Variables entered in step 1: age, gender, smoking, drinking, physical activity, BMI/WC, TCH, TG, HDLC, LDLC, SBP and DBP. HOMA-IR, homeostasis model assessment of insulin; BMI, body mass index; WC, waist circumference; TG, triglyceride; LDLC, low-density lipoprotein cholesterol.

Table S6. Baseline of demographic and clinical characteristics of subjects

in the second part of study

| Variable | T2D  (n=95) | Control  (n=95) | *P* |
| --- | --- | --- | --- |
| Age(year) | 52.43 ± 6.97 | 52.43 ± 6.97 | 1.000^c^ |
| Gender(male/female) | 52/43 | 52/43 | - |
| BMI (kg/m^2^) | 27.53 ± 3.97 | 26.37 ± 2.81 | 0.021^a^ |
| WC (cm) | 88.87 ± 9.60 | 84.29 ± 7.84 | 0.002^a^ |
| SBP (mmHg) | 128.16 ± 15.09 | 126.03 ± 13.49 | 0.307^a^ |
| DBP (mmHg) | 77.04 ± 11.05 | 76.38 ± 9.31 | 0.564^c^ |
| TC (mmol/L) | 4.82 ± 0.87 | 4.69 ± 0.79 | 0.302^a^ |
| TG (mmol/L) | 1.94 ± 1.21 | 1.47 ± 0.74 | 0.002^c^ |
| HDLC (mmol/L) | 1.58 ± 0.36 | 1.62 ± 0.41 | 0.474^a^ |
| LDLC (mmol/L) | 3.06 ± 0.88 | 3.01 ± 069 | 0.945^c^ |
| FPG (mmol/L) | 5.42 ± 0.39 | 5.22 ± 0.41 | 0.001^a^ |
| HbA1c (%) | 5.56 ± 0.29 | 5.52 ± 0.35 | 0.306^c^ |
| hsa_circ_00111707 | 4.99 ± 1.69 | 6.21 ± 2.11 | <0.001^a^ |
| Smoking (*n*, %) | 10, 10.53 | 12, 12.63 | 0.650^b^ |
| Drinking (n, %) | 10, 10.53 | 9, 9.47 | 0.809^b^ |
| Physical activity (*n*, %) | 62, 65.26 | 68, 71.58 | 0.349^b^ |

T2D, type 2 diabetes; IFG, impaired fasting glucose; BMI, body mass index; WC, waist circumference; SBP, systolic blood pressure; DBP, diastolic blood pressure; TCH, total cholesterol; TG, triglyceride; LDLC, low-density lipoprotein cholesterol; HDLC, high-density lipoprotein cholesterol; FPG, fast plasma glucose; HbA1c, glycated haemoglobin.

^a^ Student’s t-test; ^b^ Chi-square test; ^c^ Mann-Whitney U test
